# Gender-Difference in Hair Length as Revealed by Crispr-Based Production of Long-Haired Mice with Dysfunctional FGF5 Mutations

**DOI:** 10.3390/ijms231911855

**Published:** 2022-10-06

**Authors:** Ryo Takahashi, Gou Takahashi, Yuichi Kameyama, Masahiro Sato, Masato Ohtsuka, Kenta Wada

**Affiliations:** 1 Graduate School of Bioindustry, Tokyo University of Agriculture, Abashiri 099-2493, Japan; 2 Regenerative Medicine Project, Tokyo Metropolitan Institute of Medical Science, Tokyo 156-8506, Japan; 3 Department of Genome Medicine, National Center for Child Health and Development, Tokyo 157-8535, Japan; 4 Department of Molecular Life Science, Division of Basic Medical Science and Molecular Medicine, Tokai University School of Medicine, Isehara 259-1193, Japan; 5 Center for Matrix Biology and Medicine, Graduate School of Medicine, Tokai University, Isehara 259-1193, Japan; 6 The Institute of Medical Sciences, Tokai University, Isehara 259-1193, Japan

**Keywords:** fibroblast growth factor 5, long-haired mice, gender difference, genome editing, hair follicle cycle, GONAD, indels, CRISPR/Cas9

## Abstract

Fibroblast growth factor 5 (FGF5) is an important molecule required for the transition from anagen to catagen phase of the mammalian hair cycle. We previously reported that Syrian hamsters harboring a 1-bp deletion in the *Fgf5* gene exhibit excessive hair growth in males. Herein, we generated *Fgf5* mutant mice using genome editing via oviductal nucleic acid delivery (GONAD)/improved GONAD (*i*-GONAD), an in vivo genome editing system used to target early embryos present in the oviductal lumen, to study gender differences in hair length in mutant mice. The two lines (*Fgf5^go-malc^*), one with a 2-bp deletion (c.552_553del) and the other with a 1-bp insertion (c.552_553insA) in exon 3 of *Fgf5*, were successfully established. Each mutation was predicted to disrupt a part of the FGF domain through frameshift mutation (p.Glu184ValfsX128 or p.Glu184ArgfsX128). *Fgf5^go-malc1^* mice had heterogeneously distributed longer hairs than wild-type mice (C57BL/6J). Notably, this change was more evident in males than in females (*p* < 0.0001). Immunohistochemical analysis revealed the presence of FGF5 protein in the dermal papilla and outer root sheath of the hair follicles from C57BL/6J and *Fgf5^go-malc1^* mice. Histological analysis revealed that the prolonged anagen phase might be the cause of accelerated hair growth in *Fgf5^go-malc1^* mice.

## 1. Introduction

Variations in morphological characteristics of hairs, such as length, color, and localization, are common within species, breeds, strains, and even sexes. In mammals, hair length is regulated by the hair cycle, which consists of anagen (growth), catagen (involution), and telogen (rest) phases [1]. Fibroblast growth factor 5 (FGF5), one of the major regulators of the hair cycle, plays an important role in the transition from anagen to catagen phase by blocking the activation of dermal papilla cells [2,3]. 

Mutations in the *Fgf5* gene cause excessive hair growth in mice [4,5,6], cats [7], dogs [8], goats [9], sheep [10], donkeys [11], llamas [12], dromedaries [13], rabbits [14], and humans [15]. In mice, three null mutations have been identified as *Fgf5^go^* [5], *Fgf5^neo^* [6], and *Fgf5^go-moja^* [4]. Among these mutations, *Fgf5^go^* mice exhibited a 55% longer hair length than wild-type (WT) mice [5]. *Fgf^go-moja^* showed long and shaggy hair throughout the body, from the head to the tail root [4]. Although various types of *FGF5* mutations have been observed in domestic animals, except for mice, phenotype characteristics, such as hair length and distribution, have not been described. Most studies have revealed that *Fgf5*/*FGF5* mutations cause a long-haired phenotype through recessive inheritance. However, the long-haired phenotype caused by a missense mutation in *FGF5* is transmitted by co-dominant Mendelian inheritance in dromedaries [13]. We recently identified a 1-bp deletion in *Fgf5,* which is associated with excessive hair growth in Syrian hamsters, and found that this phenotype was more prominent in males than in females [16]. The excessive hair growth in this male-dominant long hair coat (MALC) phenotype is closely associated with the activation of androgen receptors (AR). Notably, the MALC phenotype exhibits heterogeneous long hairs, most prominent in the buttocks, which differ from those previously reported in *Fgf5*-null mice exhibiting a systemic long-haired phenotype [4,5,6]. The study also showed that marked sex differences in the MALC phenotype are due to mutations in the *Fgf5* gene [16]. However, how *Fgf5* deficiency promotes hair overgrowth via AR activation remains unknown. It is well known that androgen regulates hair growth, sebum production, and secretion in mammalian skin [10,17,18,19,20]. Zhang *et al.* [10] demonstrated that in *FGF5* knockout (KO) sheep, crosstalk between androgen and Wnt/β-catenin signaling plays a major role in increasing wool and active hair follicle density. However, whether FGF5 is tightly linked to AR expression in hair follicles remains unknown. Although the Syrian hamster is considered a suitable model animal, it is still an inferior experimental tool for studying genome information compared to mice. To reveal the possible crosstalk between FGF5 and AR, the development of animal models showing sex differences in hair growth, such as the MALC phenotype, is necessary.

Herein, we developed mice harboring mutations in the *Fgf5* gene (*Fgf5 ^go-malc^*) that mimic those found in hamsters to reproduce the MALC phenotype in mice. We performed genome editing via oviductal nucleic acid delivery (GONAD)/improved GONAD (*i*-GONAD), an in vivo genome editing system used to target early embryos present in the oviductal lumen [21,22,23,24]. GONAD/*i*-GONAD is simpler and more convenient than microinjection- or electroporation (EP)-based methods, which are now widely employed for genome editing.

## 2. Results

### 2.1. Generation of Fgf5 Mutant Mice

**Figure 1:**
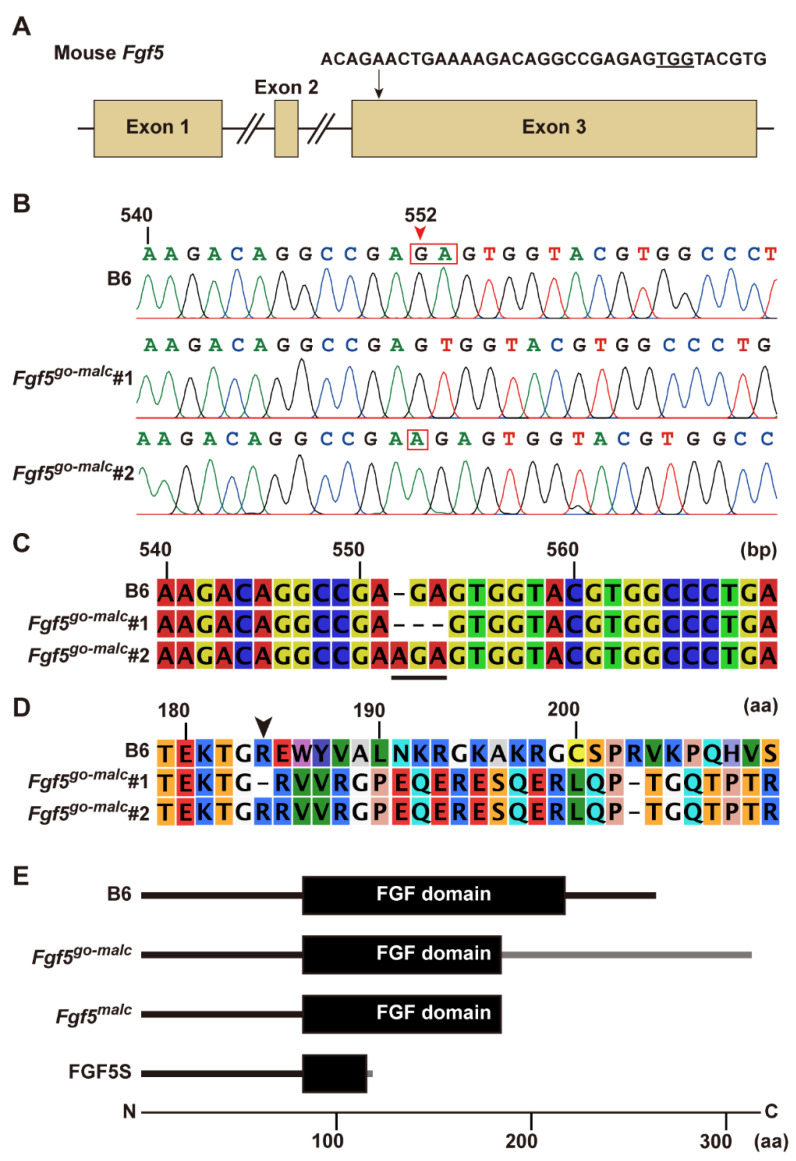
Characterization of mutations in *Fgf5^go-malc^* mice. (**A**) Sequence of the synthesized guide RNA (gRNA). The arrow indicates the gRNA target site within exon 3 of the *Fgf5* gene. The underlined sequence depicts the protospacer adjacent motif (PAM). (**B**) Electropherograms showing mutations in the two *Fgf5^go-malc^* mouse lines (*Fgf5^go-malc1^* and *Fgf5^go-malc2^*). The red arrowhead indicates indels in the *Fgf5* gene. A “GA” deletion and “A” insertion (red squares) were found in *Fgf5^go-malc1^* and *Fgf5^go-malc2^* mice, respectively. B6, C57BL/6J. (**C**) Alignment of the partial *Fgf5* cDNA sequences in the B6, *Fgf5^go-malc1^*, and *Fgf5^go-malc2^* mice. The underlined region indicates the positions of the 2-bp deletion and 1-bp insertion mutations in *Fgf5^go-malc1^* and *Fgf5^go-malc2^* mice, respectively. (**D**) Alignment of part of the predicted FGF5 amino acid sequence. The arrowhead indicates the start position of frameshift mutations that resulted in the production of abnormal or defective protein products. (**E**) Schematic representation of the wild-type (WT) and mutant FGF domains (shown by black boxes) in *Fgf5^go-malc^* mice and *Fgf5^malc^* hamsters. FGF5S is shown at the bottom. The gray line indicates the amino acid sequence of abnormal FGF5 expressed in *Fgf5^go-malc1^* mice. The mutant FGF5 protein expressed in *Fgf5^go-malc1^* mice was longer than the WT FGF5 by 48 amino acids.

A solution containing guide RNA (gRNA) and *Cas9* mRNA was injected into the oviducts of six animals at day 0.7 of pregnancy, and subsequently, in vivo EP was performed. Only one female delivered six pups, of which only one mouse exhibited a long-haired phenotype. Mating this founder mouse with WT C57BL/6J (B6) mice resulted in the generation of F_1_ progeny with a 2-bp deletion (c.552_553del) or a 1-bp insertion (c.552_553insA) in exon 3 of *Fgf5* (Figure 1B). Subsequent mating between F_1_ individuals harboring identical mutations resulted in the generation of two homozygous c.552_553del and c.552_553insA lines, all of which exhibited a long-haired phenotype. When these lines were subjected to repeated sib mating for more than five generations, the progeny reproducibly exhibited a long-haired phenotype. We termed these lines *Fgf5^go-malc1^* and *Fgf5^go-malc2^*, harboring a 2-bp deletion (c.552_553del) and a 1-bp insertion (c.552_553insA) in exon 3 of *Fgf5*, respectively (Figure 1B,C). This insertion or deletion was predicted to cause a frameshift mutation (p.Glu184ValfsX128 for *Fgf5^go-malc1^* and p.Glu184ArgfsX128 for *Fgf5^go-malc2^*), leading to the disruption of a part of the FGF domain within the FGF5 protein (Figure 1D and Figure S1). To exclude the possibility of any off-target effect in these mice, we screened for putative genomic regions showing affinity toward our gRNA using the crispor program (http://crispor.tefor.net/, accessed on 11 July 2022). Relatively higher scores were found in the introns of cannabinoid receptor 2 (*Cnr2*) and the intergenic regions on chromosomes 11 and 5 (Table S2, bold characters). These sequences were located within the non-coding genomic regions, and none of the studies have shown that mutations in genes neighboring these sequences are associated with a long-haired phenotype, and we confirmed no mutations in these regions. Although a 2-mismach count was observed in the exon region on phosphatidylinositol transfer protein cytoplasmic 1 (*Pipnc1*), its relation to hair phenotype has not been reported. Therefore, we conclude that excessive hair growth in *Fgf5^go-malc^* mice is due to mutations in *Fgf5*, which are similar to those observed in Syrian hamsters with the MALC phenotype (Figure 1E). These mutations lead to the production of the FGF5 protein with a truncated FGF domain having six abnormal amino acid residues at its C-terminal (Figure 1E and Figure S2) [16]. Since both mutations were present within the FGF domain of FGF5, both *Fgf5^go-malc1^* and *Fgf5^go-malc2^* mice shared the same phenotype.

**Figure 2:**
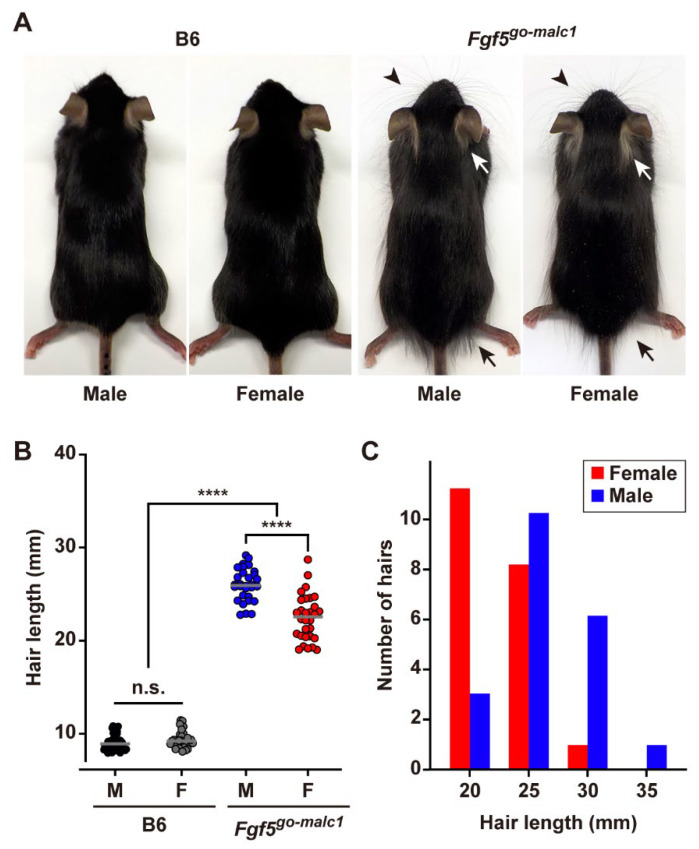
Long-haired phenotype in *Fgf5^go-malc1^* mice. (**A**) Gross appearance of the hair in B6 and *Fgf5^go-malc1^* mice. White and black arrows and arrowheads indicate representative positions of longer hairs in the neck, buttock, and whiskers, respectively. B6, C57BL/6J. (**B**) Measurement of hair length in B6 (C57BL/6J) and *Fgf5^go-malc1^* mice. Black and gray indicate hair length in male (M) and female (F) B6. Blue and red circles indicate hair length in male and female *Fgf5^go-malc1^* mice. The mice used were all littermates from B6 or *Fgf5^go-malc1^* mice. More than 30 hairs harvested from three different mice (*n* = 3) were examined and shown as hair length (mm). ****: *p* < 0.0001; n.s.: no significant differences. **(C)** Hair length distribution in the littermates [male (blue bars) and female (red bars)] of *Fgf5^go-malc1^* mice.

Indeed, *Fgf5^go-malc1^* mice exhibited a long-haired phenotype, particularly in the whisker, neck, and buttocks, when compared to WT mice (Figure 2A). Notably, the specific phenotype of the *Fgf5^go-malc^* mice was not observed in previously reported *Fgf5*-null mice [4]. Furthermore, male *Fgf5^go-malc1^* mice had longer hairs than female *Fgf5^go-malc1^* mice, which is in contrast with the WT control mice showing no apparent sex differences in hair length (Figure 2A and Figure S2). 

Next, we measured the hair length of *Fgf5^go-malc1^* and WT mice at postnatal day 53 (P53). No significant difference in the hair length was observed between WT male and female mice (9.04 ± 0.84 mm vs. 9.35 ± 0.91 mm, *p* > 0.25) (Figure 2B). Interestingly, the *Fgf5^go-malc1^* male mice had larger hairs (26.00 ± 1.74 mm) compared to female mice (22.48 ± 2.47 mm), and the difference was statistically significant (*p* < 0.0001) (Figure 2B). Notably, the hair lengths of both *Fgf5^go-malc1^* male and female mice were greater than those of WT male and female mice (*p* < 0.0001). In *Fgf5^go-malc1^* mice, females had hairs less than 35 mm long, whereas males had hairs over 35 mm long (Figure 2C). Thus, *Fgf5^go-malc1^* mice exhibited a phenotype similar to that of MALC.

### 2.2. Analysis of Mutant FGF5 Protein in Fgf5^go-malc1^ Mice

**Figure 3:**
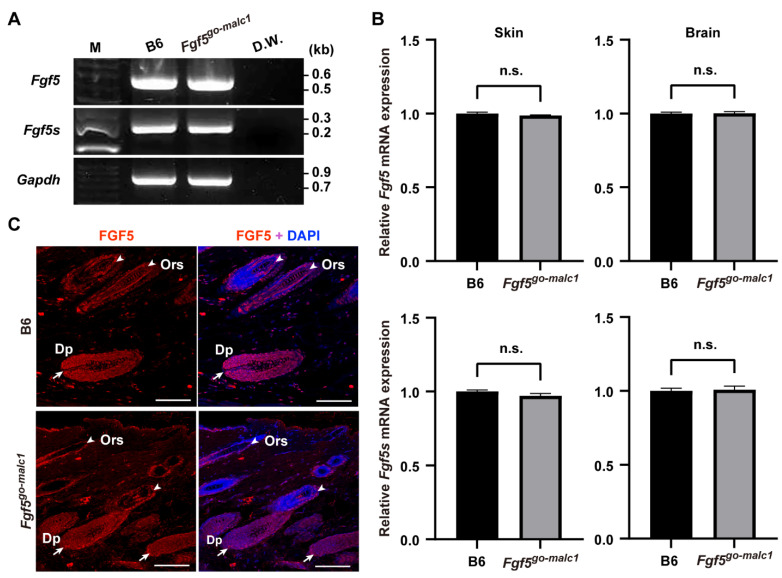
Analysis of FGF5 and FGF5S expression in *Fgf5^go-malc1^* mice. (**A**) Semi-quantitative RT-PCR analysis of *Fgf5* and *Fgf5s* transcripts in the skin. M: 100-bp DNA ladder. D.W.: distilled water. (**B**) Relative expression of *Fgf5* (upper graphs) and *Fgf5s* (bottom graphs) transcripts in the skin (left graphs) and brain (right graphs) of C57BL/6J (B6) and *Fgf5^go-malc1^* mice. Black and gray bars indicate values of relative expression levels of *Fgf5* and *Fgf5s* transcripts in B6 and *Fgf5^cr^* mice, respectively (*n* = 3; geometric means ± standard deviation). n.s.: no significant differences. (**C**) Immunohistochemical localization of FGF5 protein (red) in the skin of B6 (upper panels) and *Fgf5^go-malc1^* mice (bottom panels). Nuclei were counterstained with DAPI (blue). Arrows and arrowheads indicate the dermal papilla (Dp) and outer root sheath (Ors) in the hair follicles, respectively. Scale bar = 100 µm.

In *Fgf5^malc^* hamsters, the mRNA expression of both *Fgf5* and *Fgf5s* remains unaltered [16]. Since no appropriate antibodies against hamster FGF5 are available, it remains unclear whether FGF5 protein is indeed produced in *Fgf5^malc^* hamsters. We examined the expression of *Fgf5* mRNA and proteins in *Fgf5^go-malc1^* mice. Two alternatively spliced isoforms, *Fgf5* and *Fgf5s*, are produced from the *Fgf5* gene [2,25]. The transcript corresponding to the FGF5 protein is longer than that of the FGF5S protein. FGF5S lacks a portion corresponding to exon 2 and is composed of exon 1 and part of exon 3 [2,25]. This rearrangement was due to a frameshift and premature termination codon in *Fgf5* (Figure 1E). FGF5S lacks the FGF domain and is known to inhibit the FGF5 long isoform [26,27]. Mutations in the *Fgf5^go-malc^* mice are unlikely to affect the production of the FGF5S protein (Figure 1A,B,E). Next, we performed semi-qRT-PCR and qRT-PCR to evaluate the expression of the two *Fgf5* isoforms in the skin and brains of the different mice groups. No significant differences in the levels of *Fgf5* and *Fgf5s* mRNA were observed between WT and *Fgf5^go-malc1^* mice (Figure 3A,B). These results indicate that the production of *Fgf5* and *Fgf5s* mRNA from the *Fgf5^go-malc1^* allele is not affected by nonsense-mediated mRNA decay [28], which also appears to be true in the case of *Fgf5^malc^* [16]. Immunohistochemistry using the anti-FGF5 antibody (N-terminus) revealed that FGF5 protein is abundantly expressed in the dermal papilla (Dp) and outer root sheath (Ors) of hair follicles in WT mice (Figure 3C). A similar immunostaining pattern was observed in samples from *Fgf5^go-malc1^* mice (Figure 3C). Since the mutant FGF5 protein derived from the *Fgf5^go-malc1^* allele contains a normal N-terminus, the anti-FGF5 antibody used in this study is predicted to bind to both WT and mutant FGF5 proteins. Taken together, these results suggest that the mutant FGF5 is expressed in *Fgf5^go-malc1^* mice and its expression is localized to the hair follicles. 

### 2.3. Prolonged Anagen Phase in Fgf5^go-malc1^ Mice

*Fgf5*-null mice exhibit a long-haired phenotype due to a prolonged anagen phase in the hair cycle [29]. A similar mechanism has been reported in the *Fgf5^malc^* hamsters [16]. 

**Figure 4:**
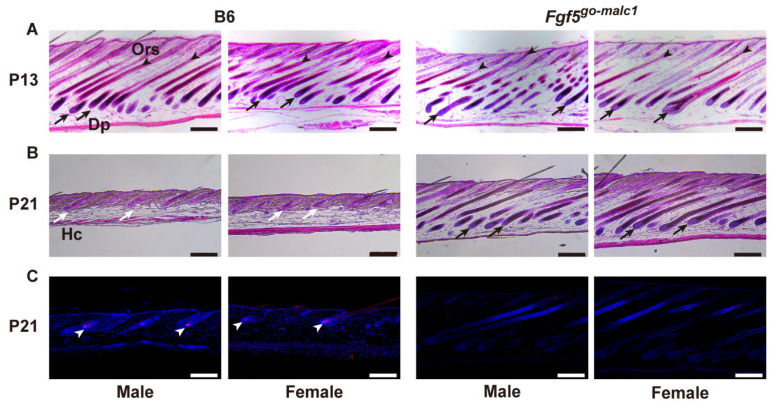
Histological evaluation of the anagen phase in *Fgf5^go-malc1^* mice. Hematoxylin-eosin (H&E)-stained skin sections from C57BL/6J (B6) (left two lanes) and *Fgf5^go-malc1^* mice (right two lanes) at P13 (**A**) and P21 (**B**). Within these two lanes, images of male and female mice are shown on the left or right side, respectively. At P21, TUNEL staining was also performed (**C**). Black arrows and arrowheads show the dermal papilla (Dp) and outer root sheath (Ors), respectively, and white arrows and arrowheads indicate hair clubs (Hc) and apoptosis cells, respectively. Scale bar = 200 µm.

To examine whether the long-haired phenotype in *Fgf5^go-malc1^* mice is also associated with a prolonged anagen phase, histological sections of the skin were prepared from WT and *Fgf5^go-malc1^* mice at P13 and P21. At P13, when the first hair cycle occurred after birth [30], the presence of Dp and Ors was discernible in both WT and *Fgf5^go-malc1^* mice, irrespective of sex, indicating that the hair cycle was in the anagen phase (Figure 4A). At P21, the skin from the WT mice showed the presence of hair clubs (Hc), which is suggestive of the telogen phase (Figure 4B, white arrows). In contrast, the skin from *Fgf5^go-malc1^* mice at P21 showed the presence of Dp in the follicles (Figure 4B, black arrows), suggesting that the anagen phase was prolonged in these mice (Figure 2B). Previous studies have shown that apoptosis is frequent in skin cells during catagen and telogen phases [16]. Next, we performed TUNEL staining to evaluate apoptotic cells for a more precise determination of the hair cycle phase. As expected, in the Hc of WT mice, apoptotic cells were frequently observed (Figure 4C, white arrowheads). In contrast, the number of apoptotic cells was lower in the Hc of *Fgf5^go-malc1^* mice, irrespective of sex (Figure 4C). These results indicate that *Fgf5^go-malc1^* mice have a prolonged anagen phase in the hair cycle, similar to the *Fgf5^go^* mice [29] and *Fgf5^malc^* hamsters [16].

## 3. Discussion

Herein, we performed genome editing to develop *Fgf5* mutant mice. These mice exhibited male-dominant long hair, although the phenotype was milder than that reported in a previous study on *Fgf5^malc^* hamsters [16]. In *Fgf5^malc^* hamsters, a 1-bp deletion in *Fgf5* (c.546delG) led to the production of truncated FGF5 [16]. In *Fgf5^go-malc1^* mice developed in our study, we speculated that frameshift mutations in *Fgf5* lead to partial disruption of the FGF domain, a region spanning 128 amino acids in the FGF5 protein (Figure 1). This is in contrast with the results of a previous study on *Fgf5^malc^* hamsters where mutation in the *Fgf5* gene led to the production of truncated FGF5 protein (Figure 1E and Figure S1) [16].

FGF5S acts as an FGF5 antagonist by competitively binding to FGF receptor 1 (FGFR1) [26]. Analysis of mutations in the *Fgf5* gene of *Fgf5^go-malc1^* mice and *Fgf5^malc^* hamsters revealed that these mutations had no effect on FGF5S functions (Figure 1E). Indeed, the levels of the *Fgf5* transcript and its translated protein product in the skin (follicles) remained unaltered in *Ffg5^go-malc1^* mice (Figure 3). In keratinocytes, FGF5S overexpression increases the levels of hepatic growth factor (HGF), which promotes DNA synthesis in the hair bulb and hair growth [27]. Thus, it is reasonable to speculate that dysfunctional FGF5 and not FGF5S might have affected follicle function, leading to a long-haired phenotype in mice. Although it remains to be determined whether mutant FGF5 binds to FGFR1, dysfunctional FGF5 protein could inhibit the normal hair cycle, particularly at the anagen to catagen transition stage. 

The *Fgf5^go-malc1^* mice exhibited a remarkable long-haired phenotype; however, the heterogeneous distribution of the long hairs in *Fgf5^go-malc1^* mice was different from that observed in mice with *Fgf5*-null mutations (Figure 2A,B and Figure S2). Two spontaneous *Fgf5*-null mutations have been reported in mice. One is BALB/cJ mice carrying *Fgf5^go^* alleles that exhibit long truncal hairs due to spontaneous deletion of exon 1 of the *Fgf5* gene [5]. The other is *Fgf5^go-moja^*, which was spontaneously isolated from ICR colonies and exhibits a long pelage caused by retrotransposon-mediated *Fgf5* deletion, leading to a loss of its transcription [4]. Moreover, *FGF5*^-/-^ rabbits with a 58-bp deletion within exon 1 produced via the CRISPR/Cas9-genome editing technique exhibit a systemic long-haired phenotype [14]. Importantly, the heterogeneous distribution of long hair was not reported in these *Fgf5*-null animals. Notably, the phenotype of the *Fgf5^go-malc1^* mice appears to be similar to that of animals carrying *porcupine* (p.Glu112Val) and *splinter* (p.Arg132Ter) alleles, both of which are established using the N-ethyl-N-nitrosourea (ENU)-mediated mutagenesis technique (https://mutagenetix.utsouthwestern.edu/home.cfm, accessed on 11 July 2022). In humans, the ratio of hair follicles in the anagen phase to those in the telogen phase of the hair cycle (anagen-to-telogen follicle ratio) differs in different parts of the body. The upper arm has a lower anagen-to-telogen follicle ratio [31], whereas follicles in the scalp are mostly in the anagen phase [15,32]. *Fgf5* mutations strongly affect the growth of human eyelashes, in which 15–40% of follicles are in the anagen phase, as well as in the forearm [15,33]. Although it remains unclear why *Fgf5^go-malc1^* mice and MALC hamsters exhibit heterogeneously distributed long hairs, partial dysfunction of FGF5 and/or normal function of FGF5S proteins may be involved in this process. 

Excessive hair growth was observed in the *Fgf5^go-malc1^* mice, although the phenotype was more prominent in males than in females (Figure 2). A previous study also showed male-dominant long hair of down hair, but not guard hair, in both WT and *FGF5*^-/-^ rabbits [14]. This suggests that male-specific hormones (androgens) might be involved in regulating sex differences in hair length, as observed in *Fgf5^go-malc1^* mice. In humans, hair follicles present in different body sites, such as the face, axilla, pubis, and chest, are vulnerable to the stimulatory effect of androgens [17]. Since complete androgen insensitivity leads to hairless skin in adults, all androgen-dependent follicles require AR activation to grow hair [18]. Moreover, in humans with 5α-reductase type 2 deficiency, there is a female-specific pattern of pubic and axillary hair growth [34]. This suggests that the requirement for 5α-reductase varies among different follicle sites [18]. Therefore, male-specific hair follicles, such as those present in the beard, chest, and pubic areas, may require 5α-dihydrotestosterone, which is synthesized from testosterone by the action of 5α-reductases and has the ability to bind more strongly to AR [18]. Testosterone and 5α-dihydrotestosterone have been reported to suppress hair growth in mice and hamsters [35,36,37]. Conversely, AR activation following testosterone administration promotes excessive hair growth in female *Fgf5^malc^* hamsters [16]. It can be speculated that in *Fgf5^go-malc1^* mice, the mutant FGF5 protein harboring a partially disrupted FGF domain inhibits the transition from anagen to catagen phase of the hair cycle in 5α-dihydrotestosterone-sensitive follicles, leading to sex differences in hair length. 

Wnt/β-catenin signaling pathway plays an important role in regulating hair growth and entry into the anagen phase [38,39,40]. Dickkopf-related protein 1 (DKK1), a natural inhibitor of Wnt, strongly suppresses the Wnt/β-catenin signaling pathway [40]. Zhang et al. [9] showed that the transcript levels of 5α-reductase type 1 (*SRD5A*), *AR*, and *DKK1* were greatly reduced in *Fgf5* KO sheep, suggesting a close relationship between FGF5 signaling and AR activation. It might be possible that the MALC phenotype observed in *Fgf5^malc^* hamsters and *Fgf5^go-malc^* mice involves the regulation of SRD5A, AR, and DKK1, although further studies are required for more conclusive evidence. In cultured cells, FGF5 overexpression leads to increased levels, activation, and ligand-independent nuclear localization of AR [41]. Moreover, in the LNCap prostate cancer cell line obtained through long-term incubation in an androgen-depleted environment, FGF5 protein levels are significantly increased, and the stimulation of AR reduces FGF5 levels in these cells [41]. Therefore, in hair follicles, FGF5, and AR interact to regulate the hair cycle, which in turn confers sex differences in the long-haired phenotype.

Although the possible relationship between FGF5 and AR activation with respect to hair growth remains to be elucidated, the *Fgf5^go-malc1^* mice developed in the present study will be helpful in studying the molecular mechanism underlying hair cycle regulation and sexual dimorphism in mammalian hair length. In future research, to demonstrate the relationships between FGF5 and AR, including related molecules that are important for hair growth, comparative analysis (including measurement of the level of those proteins and their localization) between male and female WT and *Fgf5^go-malc1^* mice may be required.

## 4. Materials and Methods

### 4.1. Mice

B6 mice were purchased from Jackson Laboratory Japan (Tokyo, Japan) and used for the development of genome-edited mice. WT B6 mice were used as controls. The genome-edited mice used in this study were generated by sib mating for more than five generations. The experiments involving in vivo transfection of mouse preimplantation embryos by *i*-GONAD were accompanied by surgery (exposure of ovary/oviducts/uterus) and operation/manipulation (nucleic acid injection via the oviductal wall and in vivo EP).

### 4.2. Generation of Fgf5^go-malc^ Mice

The gRNA targeting exon 3 of the mouse *Fgf5* gene (Table S1, Figure 1A) was designed using the online design tool CRISPRdirect (http://crispr.dbcls.jp/, accessed on 5 April 2017; Tokyo, Japan) [42]. *Cas9* mRNA was prepared by in vitro transcription from its coding sequence cloned into the pBGK vector (Addgene plasmid #65796; http://n2t.net/addgene:65796, accessed on 5 April 2017; RRID: Addgene_65796) [43] using the MEGAshortscript T7 (Thermo Fisher Scientific K.K., Tokyo, Japan) and mMESSEGE mMACHINE T7 Ultra kits (Thermo Fisher Scientific K.K., Tokyo, Japan). 

For genome editing, GONAD and its modified version, called improved GONAD or *i*-GONAD, were employed [21,22,23,24]. One day before GONAD/*i*-GONAD, superovulated female mice were mated with males. The presence of a copulation plug in the vagina at noon was designated as day 0.5 of pregnancy. A solution (1-2 μL) containing 250 ng/μL of gRNA and 500 ng/μL of *Cas9* mRNA was injected into the lumen of the oviduct of a pregnant WT female on day 0.7 of pregnancy (corresponding to the late zygote stage). Then, the in vivo EP of the entire oviduct was carried out using the square-wave electroporator CUY21SC (NEPA GENE, Chiba, Japan) under conditions of 50 V for 5 s, 8 pulses. During the operation, the mice were anesthetized using isoflurane (Fujifilm Wako, Osaka, Japan).

After surgery, the pregnant females were subjected to natural delivery. The offspring were genotyped using the PCR-based method with PrimeTaq DNA polymerase (M&S TechnoSystems, Inc., Osaka, Japan) and primers listed in Table S1, according to the manufacturer’s protocol. The PCR products were subcloned into the pT7Blue-T vector (Novagen, Madison, WI, USA) and transformed into the *Escherichia coli* DH5α strain (NIPPON GENE, Tokyo, Japan). The inserted sequence was amplified with colony-direct PCR using PrimeTaq DNA polymerase (M&S TechnoSystems, Inc., Osaka, Japan) with M13 primers. The PCR products were purified using ExoSAP IT (Thermo Fisher Scientific K.K.,Tokyo, Japan) prior to DNA sequencing using the Applied Biosystems 3730xl DNA analyzer (Thermo Fisher Scientific K.K., Tokyo, Japan). Homozygous *Fgf5* mutant mice were established by mating individuals harboring identical mutations. Since the *go* allele was first identified as an *Fgf5* mutation in *angora* mice [5], we termed the genome-edited mice as *Fgf5^go-malc1^* and *Fgf5^go-malc2^*. All experiments were performed on the offspring of *Fgf5^go-malc1^* mice.

### 4.3. Measurement of Hair Length and Hair Follicles

Hairs were harvested by depilation from the buttocks of the B6 and *Fgf5^go-malc^* mice at postnatal day 53 (P53). Hairs were then placed on a glass slide, and hair length was measured using a stereomicroscope (10 hairs per individual, *n* = 3). 

### 4.4. Quantitative RT-PCR

Total RNA was extracted from the skin and brain of one-month-old WT and *Fgf5^go-malc1^* mice (*n* = 3) using TRIzol reagent (Thermo Fisher Scientific K.K., Tokyo, Japan). The isolated total RNA (~100 μg) was then treated with ~2 μg/μL DNase I (Takara Bio, Ohtsu, Japan) for 30 min at 37 °C prior to cDNA synthesis using the SuperScript VILO master mix (Thermo Fisher Scientific K.K., Tokyo, Japan). The prepared cDNA was then subjected to semi-qRT-PCR using PrimeTaq DNA polymerase (M&S TechnoSystems, Inc., Osaka, Japan) with primers for *Fgf5* and *Fgf5s* (Table S1). Moreover, qRT-PCR-based quantification was performed using GeneAce SYBR® qPCR Mix α Low ROX (NIPPON GENE) with primers for *Fgf5s* (Mm_Fgf5_1_SG, QuantiTect Primer Assay, Qiagen, Hilden, Germany) (Table S1). Glyceraldehyde-3-phosphate dehydrogenase levels were evaluated using the GAPDH primers (Mm_Gapdh_3_SG; QuantiTect Primer Assay, Qiagen, Hilden, Germany), and were used for internal normalization. Values in the WT samples were considered as 1, and fold-changes were calculated. 

### 4.5. Histological Analysis, Immunohistochemistry, and TUNEL Assay

Dorsal skin from P13 and P21 mice were dissected and immediately fixed using Super Fix (Kurabo, Tokyo, Japan) for 14–18 h at room temperature. The fixed samples were then dehydrated using methanol, embedded in paraffin, and sectioned (5 µm thick), as described previously [16]. The sections were then deparaffinized and stained with hematoxylin and eosin (H&E).

Immunostaining was performed as described previously [44,45,46] using the anti-FGF5 antibody (#sc-376264; Santa Cruz Biotechnology, Inc., Dallas, TX, USA) and Alexa Fluor 488-conjugated goat anti-mouse IgG (#A11001; Thermo Fisher Scientific K.K., Tokyo, Japan).

TUNEL assay was performed using an in situ apoptosis detection kit (Takara Bio, Kusastsu, Japan) according to the manufacturer’s protocol. Fluorescent images were obtained using a confocal laser scanning microscope (TCS SP5; Leica Microsystems, Tokyo, Japan).

### 4.6. Statistical Analysis

Differences in hair length among groups were analyzed using two-way analysis of variance (ANOVA) with Tukey’s multiple comparison test. The analysis was performed using GraphPad Prism 9 software (GraphPad Software, San Diego, CA, USA). Differences in relative expression levels were analyzed by Welch’s *t*-test using GraphPad Prism 9 software.

## Data Availability

All data are available from the authors upon reasonable request.

## Supplementary Materials

The following supporting information can be downloaded at: https://www.mdpi.com/article/10.3390/ijms231911855/s1, Figure S1: Comparison of amino acid sequences of the FGF5 proteins from C57BL/6J (B6), *Fgf5^go-malc1^*, *Fgf5^go-malc2^* mice, and *Fgf5^malc^* hamsters; Figure S2: Differences in hair length between male and female in *Fgf5^go-malc1^* mice at P40. The analyzed mice were littermates.; Table S1: Sequences of gRNA (A), and primers used for genotyping (B) and RT-PCR (C); Table S2: Putative offtarget genomic regions of guide RNA for *Fgf5* genome-editing inferred from the crispor program (http://crispor.tefor.net/).
