# Supplementary material for: Gender-Difference in Hair Length as Revealed by Crispr-Based Production of Long-Haired Mice with Dysfunctional FGF5 Mutations
Source: Int J Mol Sci. 2022 Oct 6;23(19):11855. doi: 10.3390/ijms231911855 (PMC9569730; doi:10.3390/ijms231911855)
Supplement: Supplementary file 1 [file ijms-23-11855-s001.zip › Takahashi_R_Figure_S1.pdf]

Takahashi et al. Supplimentary Figure S1

	1 … 60
B6	MSLSLLFLIFCSHLIHSAWAHGEKRLTPEGQPAPPRNPGDSSGSRGRSSATFSSSSASSP
Fgf5$^{go-malc}$#1	MSLSLLFLIFCSHLIHSAWAHGEKRLTPEGQPAPPRNPGDSSGSRGRSSATFSSSSASSP
Fgf5$^{go-malc}$#2	MSLSLLFLIFCSHLIHSAWAHGEKRLTPEGQPAPPRNPGDSSGSRGRSSATFSSSSASSP
Fgf5^{malc}	MSLSLLFLLFCSHLILSAWAHGEKRLTPEGQPAAPRNPGDSSGSRGRSSTTSSSSSASSP

	61 … 120
B6	VAASPGSQGSGSEHSSFQWSPSGRRTGSLYCRVGIGFHLQIYPDGKVNGSHEASVLSILE
Fgf5$^{go-malc}$#1	VAASPGSQGSGSEHSSFQWSPSGRRTGSLYCRVGIGFHLQIYPDGKVNGSHEASVLSILE
Fgf5$^{go-malc}$#2	VAASPGSQGSGSEHSSFQWSPSGRRTGSLYCRVGIGFHLQIYPDGKVNGSHEASVLSILE
Fgf5^{malc}	VAASPGSQGSSPEHSSFQWSPSGRRTGSLYCRVGIGFHLQIYPDGKVNGSHEASMLSILE

FGF domain →

	121 … 180
B6	IFAVSQGIVGIRGVFSNKFLAMSKKGKLHASAKFTDDCKFRERFQENSYNTYASAIHRTE
Fgf5$^{go-malc}$#1	IFAVSQGIVGIRGVFSNKFLAMSKKGKLHASAKFTDDCKFRERFQENSYNTYASAIHRTE
Fgf5$^{go-malc}$#2	IFAVSQGIVGIRGVFSNKFLAMSKKGKLHASAKFTDDCKFRERFQENSYNTYASAIHRTE
Fgf5^{malc}	IFAVSQGIVGIRGVFSNKFLAMSKKGKLHASAKFTDDCKFRERFQENSYNTYASAIHRTE

	181 … 240
B6	KTGREWYVALNKRGKAKRGCSPRVKPQHVSTHFLPRFKQSEQPELSFTVTVPEKKKPPVK
Fgf5$^{go-malc}$#1	KTG-RVVRGPEQERESQERLQPTGQTPTRLHPLPTQVQAVRATGTFLHRHCSRKEKATGE
Fgf5$^{go-malc}$#2	KTGRRVVRGPEQERESQERLQPTGQTPTRLHPLPTQVQAVRATGTFLHRHCSRKEKATGE
Fgf5^{malc}	KTGGSGTWP*--

	241 … 300
B6	PKVPLSQPRRSPSPVKYRLKFRFG*-----------------------------------
Fgf5$^{go-malc}$#1	TKGAPVAASQKSQPSEVQTEVSLWMMLVHPGLVGNNSLYRRHRSPEGNSDTASLCCAYTC
Fgf5$^{go-malc}$#2	TKGAPVAASQKSQPSEVQTEVSLWMMLVHPGLVGNNSLYRRHRSPEGNSDTASLCCAYTC
Fgf5^{malc}	--

	301
B6	------------
Fgf5$^{go-malc}$#1	GSQVILFRFDWN*
Fgf5$^{go-malc}$#2	GSQVILFRFDWN*
Fgf5^{malc}	------------
