# Supplementary figures and images for: Gender-Difference in Hair Length as Revealed by Crispr-Based Production of Long-Haired Mice with Dysfunctional FGF5 Mutations
Source: Int J Mol Sci. 2022 Oct 6;23(19):11855. doi: 10.3390/ijms231911855 (PMC9569730; doi:10.3390/ijms231911855)

Male

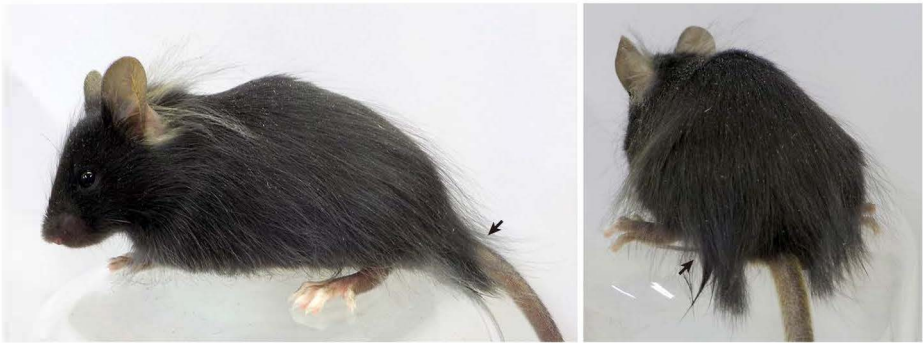

Female

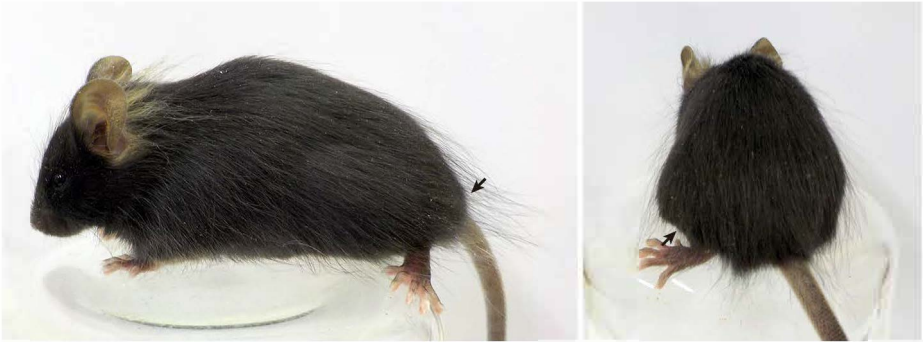

Supplement: Supplementary file 1 [file ijms-23-11855-s001.zip › Takahashi_R_Figure_S2.pdf]
